# Study of the Microstructure, Mechanical, and Magnetic Properties of LaFe_11.6_Si_1.4_H_y_/Bi Magnetocaloric Composites

**DOI:** 10.3390/ma11060943

**Published:** 2018-06-04

**Authors:** Zhengang Liu, Qiming Wu, Naikun Sun, Zan Ding, Lingwei Li

**Affiliations:** 1 Key Laboratory of Electromagnetic Processing of Materials (Ministry of Education), Northeastern University, Shenyang 110819, China; 13390156768@163.com (Z.L.); wqm331923@163.com (Q.W.); 18623859807@163.com (Z.D.); 2 Institute of Materials Physics and Chemistry, School of Materials Science and Engineering, Northeastern University, Shenyang 110819, China; 3 School of Science, Shenyang Ligong University, Shenyang 110159, China; naikunsun@163.com

**Keywords:** mechanical properties, magnetocaloric effect, LaFe_11.6_Si_1.4_H_y_/Bi composites, magnetic properties

## Abstract

We have successfully synthesized LaFe_11.6_Si_1.4_H_y_/Bi composites by cold pressing together with vacuum annealing technology, and systematically investigated the microstructure, magnetism, mechanical performance, and magnetocaloric properties. LaFe_11.6_Si_1.4_H_y_ particles are well surrounded by metallic Bi, without the formation of new phase. The maximum values of the volumetric magnetic entropy change -ΔS_M_ are as high as 51, 49, and 35 mJ/cm^3^K around 263 K, for the composites with 5, 10 and 15 wt % Bi contents, respectively. The maximum value of the compressive strength for LaFe_11.6_Si_1.4_H_y_/Bi composites increased continuously from 155 to 358 MPa with increasing Bi content, from 0 to 15 wt %.

## 1. Introduction

Magnetic refrigeration based on the magnetocaloric effect (MCE) has been considered as a new type of refrigeration system, which is environmentally friendly, energy-saving and highly efficient [1]. It is of great importance to examine materials with a large MCE that may work at different temperature ranges, especially for around room temperature [1,2,3,4,5,6,7,8,9,10,11,12,13,14,15,16,17,18,19,20,21,22,23,24,25,26]. Early studies indicated that high-purity Gd shows considerable MCE near room temperature, with nice mechanical properties [11,12]. However, its application is limited by its non-adjustable Curie temperature (*T_C_*) and high price. In order to find materials which are more favorable than pure Gd, many room temperature magnetocaloric materials have been studied, including Gd-Si-Ge, La-Fe-Si, and MnAs-based alloys [13,14,15]. One of the most promising room temperature magnetic refrigerants is La(Fe_x_Si_1−x_)_13_ hydride [11]. However, the inherent brittleness and hydrogen cracking of the 1:13 phase during the hydrogenation break the hydrides into powder, which is not in conformity with the requirements of shape for use in magnetocaloric regenerators [9,10,16]. Lyubina et al. obtained porous La(Fe,Si)_13_ materials by hot pressed (HP) La-Fe-Si particles which exhibited better mechanical properties and lower hysteresis compared with bulk materials [10]. However, when the temperature is higher than 500 °C, the precipitation of the second phase α-Fe decreases the MCE. On the other hand, the temperature of HP exceeds the dehydrogenation temperature of La(Fe,Si)_13_ hydrides. La(Fe_x_Si_1−x_)_13_H_y_ used in refrigeration equipment should have both a large MCE working temperature near room temperature, great mechanical properties, and higher thermal conductivity [17]. Nevertheless, La(Fe_x_Si_1−x_)_13_H_y_ powder could be greatly improvemed, in terms of its mechanical properties, by polymer bonding [18,19,20,21]. Heat exchangers made of polymer bonded composites combine the magnetocaloric properties of La(Fe,Mn,Si)_13_H_x_ loose powders with better mechanical and chemical stabilities. A major disadvantage of using polymeric binders to consolidate magnetocaloric alloy powders is the low thermal conductivity of composites. High thermal conductivity is a prerequisite for ensuring that magnetocaloric materials can be used. Compared with polymer bonding, methods that use a metal or alloy as a binder yield better mechanical properties and thermal conductivities [22,23,24]. If the mixed powder is compressed at low temperature, the formation of α-Fe could be avoided. Therefore, a good binder for the compression molding of La(Fe,Si)_13_H_y_ powder should have high thermal conductivity and a low softening point/melting point. Although Zhang et al. prepared a LaFe_11.6_Si_1.4_H_y_/Sn composite which demonstrated good magnetocaloric and mechanical properties compared with others [25], it should be considered that the phase transition of Sn near 286 K reduces the mechanical stability of the composite [26], which is very unfavorable for practical use. Therefore, we chose metallic Bi as a binder, and investigated the mechanical and magnetocaloric performance of LaFe_11.6_Si_1.4_H_y_/Bi composites.

## 2. Materials and Methods

The LaFe_11.6_Si_1.4_ alloy was prepared by arc melting, and then annealed in a vacuum at 1050 °C for 7 days. The annealed samples were quenched in water, and then milled. The ingots were broken and ground into powders, and the powder with particle sizes of 50–75 μm was selected by sieving, and then annealed at 423 K for 2 h with H_2_ at a pressure of 2 MPa. The hydrogen concentration of LaFe_11.6_Si_1.4_H_y_ was determined to be ~1.4 by weighing the powder before and after hydrogenation. Commercial Bi powder with the size of 10–20 μm was selected, and the LaFe_11.6_Si_1.4_H_1.4_ powder was uniformly mixed with metallic Bi powder as a binder. The mass ratio of the Bi powder in the mixed powder was 5, 10, and 15%. The mixed powder was pressed under a pressure of 1625 MPa for 10 min. The mixture was annealed inside a stainless mold (without pressure) in a vacuum at 300 °C for 10 min, followed subsequently demolding at room temperature. A cylindrical sample of φ5 × 2.7 mm was then obtained. X-ray diffraction (XRD) (Rigaku RINT 2200 diffractometer, Malvern Panalytical, Almelo, The Netherlands) was employed to examine the phase and crystal structure. The microstructure of the material was studied using a Scanning Electron microscope (SEM) (Fesm, Zeiss & Ultra Plus, Carl Zeiss AG, Oberkochen, Germany). A compression test was performed at ambient temperature using an electronic universal testing machine (AG-Xplus100kN, Shimadzu, Kyoto, Japan) with a loading rate of 1.6 μm/s. Magnetic properties were measured using a commercially available vibrating sample magnetometer (VSM, Lakeshore, Carson, CA, USA), which was added at a high field measurement system (HFMS-9, Cryogenic, London, UK). The density of the sample was measured by the Archimedes method.

## 3. Results and Discussion

**Figure 1:**
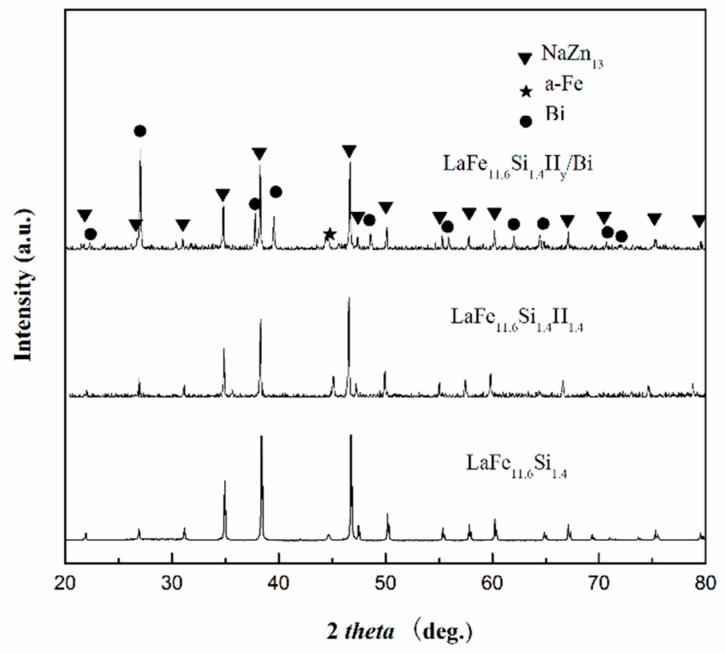
Powder XRD patterns for LaFe_11.6_Si_1.4_, LaFe_11__.6_Si_1.4_H_1.4_ alloy and LaFe_11.6_Si_1.4_H_y_/Bi samples.

**Figure 2:**
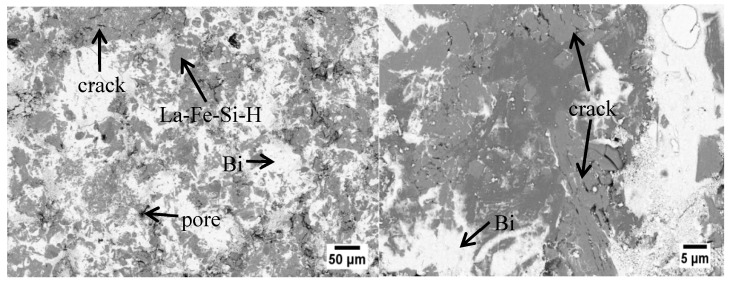
SEM images for LaFe_11.6_Si_1.4_H_y_/Bi composite with 10 wt % metallic Bi.

The phase composition and microstructure for all the LaFe_11.6_Si_1.4_H_y_/Bi composites were checked by XRD and SEM; all samples showed similar behavior. The room temperature XRD patterns and SEM images for 10 wt % metallic Bi-bonded LaFe_11.6_Si_1.4_H_y_ sample are given in Figure 1 as an example. The crystal structure includes the main phase NaZn_13_ (1:13 phase) and the secondary phase α-Fe in the matrix, in addition to the Bi phase of the binder. Compared with the lattice structure analysis of LaFe_11.6_Si_1.4_H_1.4_ and LaFe_11.6_Si_1.4_ matrix, no new phase was formed between the matrix and Bi in the final composites. The content of the secondary phase α-Fe was the same as that of the matrix. From the XRD analysis results by the Maud software, the lattice constants of the LaFe_11.6_Si_1.4_ alloy and its hydride and powder bonded sample were 1.143(2), 1.148(3), and 1.146(2), respectively, which is consistent well previously reported results [17]. This result shows that the lattice constants and unit cells volume of the hydride and bonded samples were increased. This is due to the hydrogen atoms entering the interstitial sites of the crystal lattice, which caused the lattice to expand. After processing, some of the H atoms overflow. Figure 2 show the SEM images; the gray phase is the LaFe_11.6_Si_1.4_H_y_ particle, the white area is metallic Bi, and the black area is the pores and cracks in the material. LaFe_11.6_Si_1.4_H_y_ particles are bonded together by metallic Bi. By holding the temperature above the melting point of Bi, most of the pores are filled; the metallic Bi then forms metal chains. Due to the limitation of Bi content and diffusion distance, some pores remained. It should be noted that some micro-cracks can be found on the surface of LaFe_11.6_Si_1.4_H_y_ particles, which is probably induced by the larger pressing pressure. 

For LaFe_11.6_Si_1.4_H_y_/Bi bulk composites, apparent density increases with increasing metallic Bi content, as listed in Table 1. The theoretical density of LaFe_11.6_Si_1.4_H_y_/Bi composites is the sum of the theoretical density of each component multiplied its percentages. The values of theoretical density are much higher than those of the apparent ones. All the samples have similar porosities, which is mainly caused by the same pressure. The small micro-pore content of LaFe_11.6_Si_1.4_H_y_/Bi composites would induce a significant enhancement of thermal conductivity compared with bulk.

**Figure 3:**
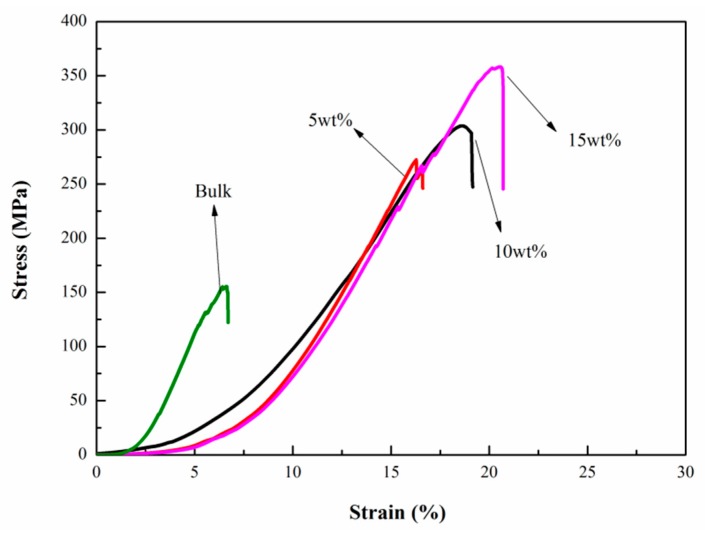
Compressive stress-strain curves for LaFe_11.6_Si_1.4_H_y_/Bi composites with 5, 10 and 15 wt % metallic Bi, respectively, in comparison with that of bulk LaFe_11.6_Si_1.4_ compound.

Figure 3 presents the stress-strain curves of the samples. Considering that powder cannot measure compressive strength, we used a bulk mother sample in this work. The maximum compressive strength of bulk LaFe_11.6_Si_1.4_ alloy is 155 MPa, which is in good agreement with previously reported values for La(Fe,Si)_13_ alloys [27,28]. The maximum values of the compressive strength for Bi powder bonded samples containing 5, 10, and 15 wt % are as high as 263, 306, and 358 MPa, respectively. Compared with the bulk sample, the maximum compressive strength of the metallic Bi bonded samples increased by about 70%, 97%, and 131%, respectively, which is much higher than that of the 3 wt % epoxy bonded LaFe_11.7_Si_1.3_C_0.2_H_1.8_ (162 MPa) [28], La_0.8_Ce_0.2_(Fe_0.95_Co_0.05_)_11.8_Si_1.2_/Sn_42_Bi_58_ (303 MPa) [29] and LaFe_11.6_Si_1.4_H_y_/Sn (170 MPa) [25]. With the increase of metallic Bi content, more boundaries between LaFe_11.6_Si_1.4_H_y_ particles will be filled, which will increase the density and enhance the binding force of the matrix particles. This evidences a good wettability between the liquid Bi and the particles. Furthermore, the toughness of pure metallic Bi is better than that of other low melting metals; thus, the sample with higher Bi content has a higher strain value. 

**Figure 4:**
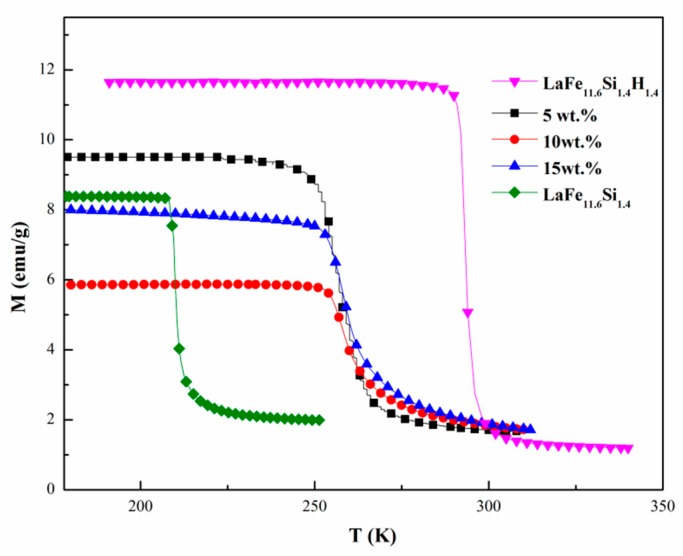
Temperature dependence of magnetization measured under 0.01 T for LaFe_11.6_Si_1.4_H_y_/Bi composites with different low-melting alloy contents and LaFe_11.6_Si_1.4_ compound.

Figure 4 shows the *M-T* curves of LaFe_11.6_Si_1.4_H_1.4_ and the bonded sample LaFe_11.6_Si_1.4_H_y_/Bi containing 5, 10, and 15 wt % metallic Bi at a magnetic field of 0.01 T. The *T*_C_ of LaFe_11.6_Si_1.4_H_1.4_ alloy is determined to be 298 K. The *T*_C_ decreases from 298 to 263 K after 1625 MPa pressure and 573 K vacuum annealing because of the partial detachment of the H atom. The transition of ferromagnetic (FM) to paramagnetic (PM) for LaFe_11.6_Si_1.4_H_1.4_/Bi bonded samples becomes slow, illustrating that the magnetic entropy change can be adversely affected by the preparation process. The magnetization (*M*) in the 5 wt % composite is higher than others, which is due to the lower content of non-magnetic phase. In contrast, *M* in 10 wt % composite is lower, which means particles can be protected by more binder during the press processing.

**Figure 5:**
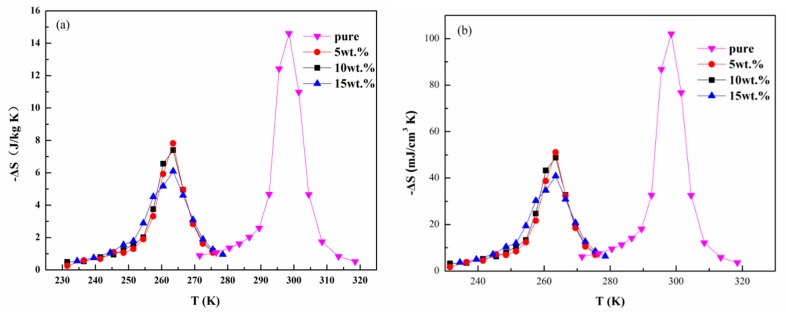
(**a**) Temperature dependence of mass Δ*S*_M_ for LaFe_11.6_Si_1.4_H_1.4_ and LaFe_11.6_Si_1.4_H_y_/Bi composites with different metallic Bi contents under a magnetic field change of 2 T; (**b**) Temperature dependences of volumetric Δ*S*_M_ for LaFe_11.6_Si_1.4_H_1.4_ and LaFe_11.6_Si_1__.4_H_y_/Bi composites with different metallic Bi contents under a magnetic field change of 2 T.

Figure 5a gives the isothermal magnetic entropy change curve of LaFe_11.6_Si_1.4_H_1.4_ and the composites with metallic Bi contents of 5, 10, and 15 wt % under an applied magnetic field change of 0–2 T. The maximum magnetic entropy change (-ΔS_M_^max^) for pure LaFe_11.6_Si_1.4_H_1.4_ is 14.6 J/kg K. In parallel, the -ΔS_M_^max^ are 7.8, 7.4, and 6.1 J/kg K for the composites with metallic Bi contents of 5, 10, and 15 wt %, respectively. Obviously, the -ΔS_M_^max^ of the composited samples shows a decreased tendency with increasing metallic Bi content, which may originate from the decrease of the NaZn_13_ phase and the cracks induced by the pressing of 1625 MPa, as observed in the SEM image. As reported in ref. [30], the smaller MCE was due to the removal of extrinsic hysteresis and the diminishing of grain boundaries of the small 1:13 particles. Figure 5b shows temperature dependence of volumetric -ΔS_M_ for pure LaFe_11.6_Si_1.4_H_1.4_ and LaFe_11.6_Si_1.4_H_y_/Bi composites with different metallic Bi contents under a magnetic field change of 0–2 T. Considering the practical application, it is necessary to give another expression of the isothermal magnetic entropy change, i.e., volumetric magnetic entropy change. It can be seen that the maximum values of the volumetric -ΔS_M_ are as high as 51, 49, and 35 mJ/cm^3^K around 263 K for the composites with 5, 10, and 15 wt % Bi contents, respectively. Although these values are lower than 102 mJ/cm^3^K for pure LaFe_11.6_Si_1.4_H_1.4_, such maxima are close to those of the LaFe_11.7_Si_1.3_C_0.2_H_1.8_ (54.7 mJ/cm^3^K) composites [28] and La(Fe,Mn,Si)_13_H_y_ (55–65 mJ/cm^3^K) bonded by the epoxy resin [31].

## 4. Conclusions

We have fabricated the LaFe_11.6_Si_1.4_H_y_/Bi magnetocaloric composites with two-phase alternating distribution microstructure by vacuum annealing after cold pressing. The crystal structure of the matrix LaFe_11.6_Si_1.4_H_1.4_ shows no disruption, and the mechanical property of the composite was improved by the continuous increase of the metallic Bi among the matrix particles. The maximum values of the compressive strength for LaFe_11.6_Si_1.4_H_y_/Bi composites with Bi contents of 5, 10, and 15 wt % are as high as 263, 306, and 358 MPa, and the corresponding volumetric -ΔS_M_^max^ are 51, 49, and 35 mJ/cm^3^K under Δ*H* of 0–2 T at 263 K, respectively. The Curie temperature decreases to 263 K after pressure and vacuum annealing. The current study may provide a rising useful way to prepare the La-Fe-Si based-magnetocaloric composites.

## Figures and Tables

**Table 1:** Density, porosity of the LaFe_11.6_Si_1.4_H_y_/Bi composites with different metallic Bi contents.

Metallic Bi Content (wt %)	Theoretical Density ρ (g/cm^3^)	Apparent Density ρ (g/cm^3^)	Porosity (%)
5	7.14	6.53	8.52
10	7.24	6.61	8.86
15	7.35	6.70	8.82
